# The Health and Safety Experiences of Precariously Employed Bangladeshi Immigrant Workers in Toronto During the COVID-19 Pandemic

**DOI:** 10.1177/10482911241239263

**Published:** 2024-03-14

**Authors:** Stephanie Premji, Momtaz Begum, Kishower Laila, Sultana Jahangir, Adam Zvric

**Affiliations:** 1 School of Labour Studies, 3710 McMaster University, Hamilton, ON, Canada; 2 Dalla Lana School of Public Health, 7938 University of Toronto, Toronto, ON, Canada; 3 South Asian Women and Immigrants’ Services, Toronto, ON, Canada

**Keywords:** COVID-19 pandemic, precarious work, occupational health, immigration

## Abstract

Racialized immigrants in Canada have been disproportionately impacted by the COVID-19 pandemic. Our qualitative, community-based study with South Asian Women and Immigrants’ Services examined the impact of the second and third waves of the pandemic on the work and health of precariously employed Bangladeshi immigrant women and men in Toronto. Our study is based on interviews and focus group discussions with 45 workers, all conducted in Bangla, and 11 key informants. Interviews reveal work transitions, an increase in precarity, work in essential sectors, exposures at work, home and in transit, workplace prevention and management gaps, and an inability to take time off, with significant impacts on workers’ physical and mental health. We discuss the implications of our findings for prevention, preparedness, and response by workplaces and governments to decrease the risk and reduce the impact of infectious diseases emergencies in the precarious work sector.

## Introduction

In Canada and around the world, the impact of the COVID-19 pandemic has been disproportionately borne by women and members of racialized and immigrant communities.^1–3^ For instance, as of December 31, 2021,^a^ racialized communities in Toronto accounted for 69 percent of all COVID-19 cases while making up 52 percent of the population.^4^ While public and political discourse has largely individualized the causes of and solutions for inequities,^5^ research has linked COVID-19 infection and death rates to structural inequities, including inequities in work.^6,7^ Evidence shows that racialized and immigrant workers are overrepresented in businesses or services that were designated as “essential” during the pandemic^8,9^ such as care (services that involve close contact with people, such as health care and childcare), manufacturing, warehousing and construction, where risk of infection was increased relative to other sectors.^10^ Inequities also exist within sectors. For example, within health care the COVID-19 infection rate increases as occupational status decreases.^11^ Similarly, the representation of racialized women increases as status decreases.^12^

At the same time, racialized immigrant workers are disproportionately found in precarious employment.^13^ Precarious work is particularly prevalent in certain sectors, such as retail, food services and accommodations, child care, agriculture, and construction,^14^ which were largely classified as essential during the pandemic.^15^ Studies have documented greater job loss and insecurity for those in precarious work during the pandemic,^16,17^ and negative impacts of both working and employment conditions on physical and mental health and well-being.^18,19^ The combination of low wage and lack of, or inadequate, access to benefits was also found to limit precariously employed workers’ ability to take time off work for pandemic-related reasons.^20,21^ Statistics Canada survey data indicate that 74 percent of low-income workers as well as a significant majority of workers in seasonal (84 percent), casual/on-call (76 percent) and contract (67 percent) employment report no paid sick leave benefits, compared to 56 percent of workers in regular employment.^22^

However, knowledge about the mechanisms through which precarious work, within its larger socio-economic and political context, creates vulnerabilities that worsen work and health outcomes from public health crises, remains limited.^23^ Also limited is understanding how these dynamics differently impact workers at the intersection of social locations, such as racialized and immigrant status and gender.^24,25^ Our community-based study initially set out to investigate experiences of work-related injury and illness when work is not stable or secure, including experiences with employers, workers’ compensation, and return-to-work. However, as the study commenced during the early stages of the pandemic, an additional focus was to document the impact of COVID-19 on workers, within the Toronto Bangladeshi community. Our paper examines this impact in the wider context of preparedness and response by workplaces and governments, with the objective of proposing evidence-based solutions to decrease the risk and reduce the impact of infectious disease public health emergencies on precarious workers.

## Focus on the Toronto Bangladeshi Community

Our study was conducted in the Bangladeshi Community in Toronto, Ontario, the largest city in Canada and main destination for newcomers.^26^ According to the 2021 census, 7.1 percent of the Canadian population reported being of South Asian descent, 3 percent of whom were born in Bangladesh. About half of Bangladeshi immigrants (36 670) live in Toronto.^27^ The majority immigrated to Canada through the federal skilled worker program,^26^ which provides a path to permanent residence for applicants who meet certain work experience, education, and language eligibility criteria. But, while most Bangladeshi immigrants are university educated and have experience in in-demand fields,^28^ many end up in low-paid, precarious work.^29^ Studies have documented significant de-skilling among this and other racialized immigrant populations in Canada due to factors such as discrimination by employers, restrictive professional accreditation systems, language barriers, and limited professional networks.^30,31^

South Asian community members, like other racialized groups, have been disproportionately impacted by the pandemic.^4^ Explanations for inequities in the rates of COVID-19 infections, particularly in the early stages of the pandemic, emphasized “blame the victim” discourses, for instance by focusing on the role of religious celebrations like Diwali and practices such as living in multigenerational households, in driving infections.^32,33^ This is in line with what has been termed the “culturalist” discourse, which positions the source of inequities as rooted in workers’ knowledge, attributes or behaviors, a framing which may in turn lead to stereotyping and stigmatization, and to the obscuring of the social, economic, and political forces that create and amplify inequities.^34^ Indeed, while a growing body of research has documented inequities in health and safety experiences and outcomes, there exists little research on how inequities are produced and how to reduce them.^35^ Thus, several authors have argued that a structural lens is needed to uncover and address the root cause of occupational and public health inequities, including those related to the COVID-19 pandemic.^34–37^ It is from this perspective that we investigated the pandemic work experiences of Toronto Bangladeshi immigrant workers.

## The Policy Context

Our study understands micro-level experiences of work and related inequities in the context of broader economic and political conditions. Therefore, it is necessary to describe the larger policy environment at the time of our study. At the onset of the pandemic, the provincial government in Ontario declared a state of emergency and ordered all workplaces deemed non-essential to close in an effort to mitigate the spread of the SARS-CoV-2 virus.^38^ The government issued a list of workplaces that would be designated as essential, including any “[b]usinesses, facilities and services that support and facilitate the two-way movement of essential goods within integrated North American and Global supply chains.”^15^ After decades of trade liberalization, the inclusion of this criterion meant that much of the manufacturing sector in Ontario could be considered essential. Changes to Ontario's list of essential workplaces correlated with an increase in recorded lobbying efforts by businesses who sought the designation of essentiality.^39^

In response to surging levels of unemployment alongside a restrictive national employment insurance scheme, the federal government developed a series of emergency response benefits to act as direct income supports to those facing unemployment, the most notable among them being the Canadian Emergency Response Benefit (CERB). The widely accessed benefit provided eligible workers who were forced to stop working, either by workplace closure or as a result of a caregiver role, or whose working hours were reduced due to COVID-19, with $2,000 a month. However, workers were ineligible if they quit their job voluntarily, earned < $5,000 in 2019 or in the 12 months prior to their application, or > $1,000 in income for the weeks in which they applied.^40^ This benefit design automatically excluded workers designated as essential whose workplace was not forced to shut down, including many in positions characterized by low-wages and precarity and, as such, these workers were required to choose between continuing to work in-person or forgo any source of income. The CERB was ended in September 2020, roughly coinciding with the start of this study, and replaced by a series of similar government benefits with stricter eligibility requirements and less generous replacement income levels.^b^

For those required to work during the pandemic, the Ontario government, together with local public health units, did mandate new health and safety measures, the requirement of personal protective equipment (PPE) in the workplace, and increased the number and frequency of health and safety inspections.^41–43^ However, whether employers were required to provide PPE free of charge was unspecified,^44,45^ and inspections focused largely on educating employers rather than enforcement.^42^ Employers were advised, though not mandated, to utilize the hierarchy of controls, with (a) engineering measures such as good ventilation as the most effective, followed by (b) administrative measures such as screening, sanitization, maintaining physical distance, and (c) use of PPE.^46,47^

Furthermore, recognizing the contradiction between the value of their work and the often-low wages of essential workers, an agreement between the federal and provincial governments in April 2020 sought to provide a temporary pay increase for essential workers, with the federal government providing a majority of the funding and the provincial government determining eligibility and administering the program.^48^ In Ontario, this pay increase was limited to workplaces in health care, long-term care, retirement homes, social services, and corrections only. This excluded many low-wage workers deemed essential and yet working in-person in workplaces outside of those designated as eligible.^49^ This temporary pay increase ended for most eligible essential workers in August 2020, when interviews for this research project began.

Finally, at the outset of the pandemic, in Ontario, there were no provincial government-mandated paid sick days. When presenting symptoms of COVID-19, workers in Ontario were encouraged to apply for the federal Canada Recovery Sickness Benefit, which provided $500 per week for up to a total of six weeks starting from September 2020.^50^ However, the application and processing period meant that there was a gap between taking time off from work due to COVID-19 and receiving benefit payments, presenting a non-option for workers living paycheque-to-paycheque. It was not until the Spring of 2021 that, under intense pressure, the Ontario government implemented a temporary provincial program to provide workers with three government-funded paid sick days.^51^ This coincided with the latter part of interviews conducted for this study.

## Methods

This study was co-led by a McMaster University researcher and South Asian Women and Immigrants’ Services (SAWIS),^c^ a small nonprofit organization that is run by and works primarily with Bangladeshi women in the Toronto neighborhood of Oakridge-Crescent Town. A community-engaged approach was adopted whereby McMaster and SAWIS were equally involved in all aspects of the research process, including the design, analysis, and dissemination of the data. The project was conducted by peer researchers, Bangladeshi women with lived experience of precarious employment. Indeed, given the nature of research procedures, timelines and funding, employment on our research team was also precarious (time-limited contract, sporadic work hours, etc.). Throughout the research process, we provided research training and support to peer researchers with the objective of building research capacity at the community level.^d^

Between August 2020 and August 2021, a period which overlapped with the second and third waves of the COVID-19 pandemic in Ontario, we conducted 31 semi-structured interviews with workers from the Toronto Bangladeshi community and four focus group discussions (14 participants total, 3-4 in each group). The samples included workers who were currently working or had last worked in a precarious job and had experienced a physical and/or mental illness or injury due to work. Precarious work was defined as insecure or unstable employment, such as work that is low-waged, temporary, gig-based, on-call, involuntarily part-time, or informal. In line with the Law Commission of Ontario, we posited low wage as a necessary and sufficient condition of precarious employment.^52^ Workers were recruited through SAWIS contact lists, Facebook groups for the Toronto Bangladeshi community, and snowball sampling. Workers were asked in part about their labour market and work experiences during the pandemic and how these shaped, and were shaped by, their health and safety. In addition, eleven key informants with knowledge of the Toronto Bangladeshi community, and who were identified through our networks and snowball sampling, were individually interviewed. Key informants were asked about trends they observed in the work and health experiences of community members during the pandemic.

Interviews and focus group discussions each lasted around 2 h, were conducted remotely by phone or Zoom, and audio recorded. Workers were compensated for their participation with a $45 honorarium. Prior to data collection, all participants were provided with a consent form. Worker consent forms were available in English and Bangla. Consent was obtained in writing, or verbally and recorded in a consent log. All interviews with key informants were conducted in English, while all worker interviews were conducted in Bangla. Bangla audio files were translated into English and transcribed in a single step by peer researchers who had high proficiency in both languages. The decision to translate data prior to analysis was made to ensure all team members could participate in the analysis. Using NVivo software, interview transcripts were each coded by a team member using a mixed coding strategy, whereby some codes were defined a priori while others emerged from the data. Team members worked collectively on the coding framework and on the development and analysis of themes. Ethics approval (no. 1732) was obtained from the McMaster Research Ethics Board. All names are pseudonyms and details have been changed as appropriate to protect the anonymity of participants.

Our research is informed by political economy (PE),^53^ a framework that contributes to understanding why different stakeholders benefit differently.^53,54^ A PE lens recognizes the influence, on micro-level experiences, of broader systemic factors, including the rules governing prevention and compensation systems, which are in turn shaped by the power dynamics and interests of the state, employers, and workers, as well as broader political and macro-economic conditions.^54–57^ Applying a PE lens, we sought to uncover inequities within political and economic systems, and reveal how policies (e.g., labour market and social welfare) influence and shape the experiences of precarious employment and resulting health inequities among our research participants.

At the same time, our project applied an equity lens to investigate the experiences of health and safety among marginalized workers employed in precarious conditions, during a public health emergency. Currently, there is a gap in our understanding of the systemic factors that contribute to health and safety inequities.^34^ Flynn et al.^58^ have argued that addressing inequities and their underlying power imbalances requires a holistic approach that accounts for the broader structures within which these patterns are produced. Our study integrated this perspective by including those who have traditionally lacked power and resources in all aspects of our project, and by emphasizing the systemic root causes of inequities in our analyses.

## Demographic Characteristics of Participants

Worker characteristics are presented in Table 1. Workers were almost equally distributed in the 31–40, 41–50, and 50 + age categories. Large majorities were women (31/45), married (38/45), and/or had children (39/45). Almost all had been in Canada for 10 years or fewer (38/45) and had immigrated under the skilled worker category (31/45), primarily as dependents. Almost all had a post - secondary education. Twenty five of the 45 hold master's degrees. Despite this, participants worked in low wage jobs in factories, retail and restaurants, child care, and security. Data on whether participants worked in formal or informal employment was not collected.

**Table 1.** Characteristics of Worker Participants.

Characteristics		Interview participants N = 31	Focus group participants N = 14	Total workers N = 45
Sex	Men	11	3	14
	Women	20	11	31
Age	18–30	2	2	4
	31–40	10	4	14
	41–50	12	3	15
	50 +	7	5	12
No. of children	1–2	22	9	31
	3 or more	5	3	8
	None	4	2	6
Years spent in Canada	5 years or less	9	6	15
	6–10	16	7	23
	11–25	6	1	7
Immigration category	Skilled principal	5	4	9
	Skilled dependent^a^	16	6	22
	Investor	1	0	1
	Family	2	1	3
	Refugee	4	3	7
	Temporary work permit	2	0	2
	Did not answer	1	0	1
Education/training	High school or less	1	3	4
	College	4	0	4
	Bachelor	10	1	11
	Master's	15	10	25
	Did not answer	1	0	1
Occupation^b^	Child care	7	2	9
	Factory	11	5	16
	Security	2	1	3
	Retail & restaurant	11	5	16
	Office	0	1	1
Marital status	Married	28	10	38
	Other^c^	3	4	7

a Dependents include spouses or children.

b Occupation represents the current or last sector (for those who were not working) at the time of the interview.

c Other include single, divorced, separated, widowed, and engaged.

Key informant affiliations are presented in Table 2. They included representatives from community organizations, employment services, unions, government, and the media.

**Table 2.** Affiliations of Key Informant Participants.

Affiliation	Number of key informants
Community organizations (incl. community legal clinics)	6
Employment services	1
Media	1
Government (public officials)	2
Union	1
Total	11

## Results

Our results indicate that workers’ pandemic work experiences were characterized by transitions in and out of employment, increased employment and financial precarity, work in jobs designated as essential, COVID-19 exposures at work, home and in transit, workplace prevention and management gaps, inability to take time off, and physical and mental health impacts. We present these findings below.

### Transitioning In and Out of Employment

At the time of interview, workers’ employment status varied. Some had stopped working before the pandemic (10/45), in some cases due to workplace injuries. Others had quit working during the pandemic because of health concerns or child care needs (10/45) or had stopped due to permanent or temporary layoffs (12/45). While some of the workers who were not currently working were actively looking for work, others had paused their search. Women, more so than men, found their work interrupted by the pandemic. Reported reasons were caring responsibilities, health issues, and fear of contracting or transmitting the SARS-CoV-2 virus.

The remaining workers, 6 men and 7 women, were working at the time of interview. For some, the pandemic generated new opportunities for finding work or changing jobs. Others explained that they did not want to work but had no choice as they were designated essential or otherwise ineligible for government benefits. For example, Romena, a 48-year-old factory worker who was job searching at the time of interview, explained:

It is not possible to maintain distance between two people in a production line in our workplace. Workers like me cannot think of COVID, because I did not have that eligibility yet to receive COVID benefit from the government like some other people. I will not receive any benefit from the government. But, I have to live my life. For that, I have to work.

Key informants explained that difficulties navigating benefit systems and delays in obtaining benefits also played a role in workers continuing to work. As well, they pointed to the inadequacy of emergency government benefit amounts relative to the high cost of living in Toronto. Workers and key informants also reported pressure to return or continue to work by employers under the explicit or implied threat of losing job, income, or access to benefits. Jerry, a community organizer, explained how women who had children to care for during school closures experienced pressure to return to work:

They’re not accommodating the women who have to stay home because when the schools are closed, for example, we had employers with all women, the women would report that they have to stay home because they can’t come to work because their kids are home, because schools were closed. And employers told the women that if you don’t come to work, we’ll put down on your record of employment that you quit [and were therefore ineligible for benefits].

Importantly, workers’ employment situation was fluid over the initial waves of the pandemic as they reported transitioning in and out of employment in response to changing conditions.

### Increased Employment and Financial Precarity

Many workers reported an intensification of the precarity of their employment and finances during the pandemic, for instance as they moved into gig or on-call work or experienced intermittent employment or a reduction in work hours. In some cases, permanent positions were replaced with temporary agency employment. Abid, a 51-year-old security guard reported:

I started a job as security [guard]. I continued there for approximately six months … After six months, they suddenly transferred me. They told me “We are transferring you somewhere else. We won’t keep you in this hospital.” I replied, “It's okay.” But they actually did not transfer me. They rather made the job on-call basis.

The jobs held by participants were not subject to pandemic pay increases despite the additional risk and cost, such as having to purchase PPE due to the law's failure to specify whether the employer was required to pay for it, as indicated above. For those who had stopped working, emergency government income supports were seen positively though, as noted above, there were concerns about benefits levels, access, and eligibility. Some workers reported financial hardship because emergency benefits ended before they were able to return to work. They were often pushed to other, less advantageous, benefits. These benefits were stricter in their eligibility requirements, required re-application more frequently, entailed a lag between applying and receiving benefits, and/or had a lower benefit payout.^59^ Participants reported significant financial strain as families struggled to pay for necessities. Rachel, a public official and a key informant for this study, explained that due to both the increase in economic precarity and the high cost of rent, “a great many members of the Bangladeshi community are now facing eviction.”

### Work in Jobs Designated as Essential

Many participants were employed in work designated as essential. Workers and key informants contested the broad application of the designation. Tania, a 40-year-old coffee shop worker, explained how keeping some arguably nonessential workplaces opened during the pandemic unnecessarily put workers at risk:

However, this recent lockdown did not restrict people from entering the coffee shops. People are going one after another in our shop. But we are not sure who is COVID positive. I think COVID is getting worse due to these reasons. A shutdown should literally shutdown everything. Why did they not close these coffee shops like others?

Participants also reported a lack of government enforcement to shut down workplaces that were not designated essential or that exploited the designation. Workers described the contradiction of being designated essential while being treated as expendable. For Nahida, a 34-year-old factory worker, this expendability was manifested through vaccination policies that did not prioritize all essential workers equally: “I would say that we are exposed to COVID risk since the beginning. The government should have vaccinated the essential workers first.” In Ontario, the phased rollout of the COVID-19 vaccination campaign saw health care workers and workers in long-term care and retirement homes prioritized under Phase One, among other defined vulnerable populations not categorized by work.^60^ Other workers who could not work from home were not eligible to receive the vaccine until Phase 2, most included in the latter stages of this phase.^61^

### COVID-19 Exposures at Work, Home and in Transit

COVID-19 exposed workers to new health and safety risks on top of those that already existed in their workplaces. Among the seven worker participants who contracted COVID-19, six believed that their workplace was the source of infection. The impossibility of physically distancing in many workplaces, such as on the factory floor, was a major concern. Participants also described crowding at certain times (i.e., shift start and end times and lunch breaks) and places (i.e., lunchroom, washroom, and water fountains). Certain workplace policies and practices increased COVID-19 risk by encouraging mixing and crowding. For example, participants reported that during busy periods, some workplaces joined employees from different shifts without maintaining proper health and safety protocols. In client-facing jobs, workers similarly reported exposure to COVID-19, particularly as some customers or clients did not follow COVID-19 regulations or guidelines, and not all guidelines were adequately protective.

In addition to workplace risks, most workers were living in overcrowded high-rise buildings and were commuting on crowded public transit where mask-wearing and physical distancing was inconsistent and would have done very little to prevent disease transmission in the absence of adequate ventilation. Nazia, a community organizer, described how guidance to physically distance was not realistic for workers in these settings:

He needs to go to work by 10 and he's waiting for the elevator for 10 minutes and he doesn’t care about social distancing; he just needs to get into the elevator and in there are more people. Other is bus and bus it's written “OK one seat is empty blah blah blah … and you should maintain … [social distancing]” but during the office hours I have seen myself, during the office hours, nobody cares about this social distancing because they cannot, they need to go, right.

Some workers sought alternatives to public transit, such as walking or ride sharing, but choices made to protect oneself from COVID-19 sometimes came at a cost, financial, or otherwise. Shoma, a 33-year-old coffee shop worker, explained how trying to reduce her exposure to COVID-19 led her to walk long distances, and how this health burden combined with existing workplace exposures:

It is required to maintain distance in a bus. But, so many people board a bus that you cannot even insert a needle between two people. I work 8 hours in a row without any washroom break or normal break and after that I walk 1 hour and 35 minutes to reach home. My feet got bruised. I did it, so that my baby stays healthy.

### COVID-19 Workplace Prevention Gaps

According to workers, screening for COVID-19, when present in the workplace, consisted of taking workers’ temperature or self-screening on a common device. Several workers felt that the screening process itself posed an infection risk by encouraging crowding at entry points. Other prevention measures were reported to be unevenly applied or lacking. While partitions were installed in some factories (despite the fact they may not be appropriate in this setting, particularly in the absence of ventilation^62^), several workers reported that these were installed only in the front production lines visible to visitors, suggesting that they were used as a form of COVID theater. Workers also described an absence of sanitization of common spaces and a lack of adequate PPE. Security guard Abid, who contracted COVID-19 after working at the entrance of a hospital where he regularly screened COVID-19 patients, explained how the provision of PPE varied among essential workers:

They did not give us the personal protective equipment as they did for the nurses. We just had the security uniform and nothing else. Besides that, we had face shields and masks.

Several workers discussed the complete absence of prevention measures in their workplace and the individualized approach to prevention, as they obtained their own PPE (because the law did not clearly require employers to pay for it) and did their best to follow public health guidelines. Key informants described workers being unable to afford high quality PPE and turning to less effective options such as reusable cloth masks, and/or reusing disposable PPE. They also explained that workers were unable to speak up against unsafe practices because, when they did, they were told to go home. In contrast, the presence of infection prevention measures was reported in workplaces that involved a public-facing component, such as child care settings. Jesmin, 46-year-old early child care assistant, described the prevention measures adopted by her workplace:

Yes, we received guidelines. They sent a few training modules. I did a few courses through online. They trained us very nicely, and the day care center is also maintaining all rules and regulations very nicely and carefully. I am very much happy. Little kids are coming in the centre, we all are very honest in this regard.

In some instances, employers’ attempts to minimize COVID-19 risk, for example by staggering shifts, came at the cost of lost hours and income for workers. Jahida, 33-year-old factory worker, described how this practice resulted in her getting reduced work hours.

At that time [during the pandemic], we were getting few hours because once one shift ends, another shift starts. We would leave an hour earlier and then the people in the next shift would come. This would prevent large gatherings.

### COVID-19 Workplace Management Gaps

Participants expressed concern about how workplaces handled confirmed or suspected COVID-19 cases. They noted inadequate contact tracing, as many had not been informed of positive cases or outbreaks by employers and had only found out through co-workers. Afrin, a 57-year-old restaurant worker, provided an example of how the lack of communication from her employer, combined with anxiety about contracting the virus, impacted her mental health:

All of a sudden, one day I heard that someone was absent and they gave me extra hours because of that … The first day I didn’t realize what was going on, nobody was saying anything … Second day, I heard that the person whose shift I am covering has COVID … I felt like oh Allah, they must have touched this thing, worked in this area, sat on this chair, and I am working with all the same things. I felt a huge sense of dread within myself.

Participants also discussed employers’ delayed responses upon becoming aware of new COVID-19 cases, highlighting employers’ poor communication practices. Shurovi, a 44- year-old factory worker, explained:

The line the [COVID positive] woman was from, they sent everyone in that line to quarantine only. There were most probably 15 or 17 people there, they all went into quarantine. It's a huge floor, and on that floor there are approximately 12 to 15 tables. Some machineries are attached to the tables, and workers work on both sides. [That worker had used the] same washroom. Yes, but only people from the line went into quarantine.

Despite guidelines recommending a minimum 10 days of isolation for symptomatic individuals,^e^ workers described being asked to return to work if the result of their COVID-19 test was negative, even though they might still be shedding virus when they cough. In a context of precarity and with pressure to return to work by their employer, many reported returning to work with symptoms. As well, proper quarantine and isolation were not typically feasible due to workers’ family or financial circumstances, because those having to care for others or living in crowded housing could not fully isolate from other members of the household.

### Inability to Take Time Off

Many participants talked about the financial inability to quarantine, isolate, recover, or get tested or vaccinated, despite public health messaging advising to do just that. Government policies, namely the lack of a permanent paid sick leave program, incentivized workers to work sick. Almost all workers in the study lacked access to paid sick leave. Donna, a public official and key informant, observed that the lack of paid sick leave prevented workers from following public health guidelines:

A lot of workers have just decided that they were going to go to work because “if I go through a test, then that's a day for myself, because from line-ups to getting the test to getting the result, those are a few days that I have to miss, which means I’m not getting paid.”

Power imbalances in the precarious work sector that pre-dated the COVID-19 pandemic also contributed to workers’ inability to take time off. Jerry, a community organizer, explained that access to paid sick leave is not sufficient to guarantee workers’ ability to stay home when sick:

There's no sick days paid or unpaid. There's like “if you don't come to work you’re fired.” I mean, yeah, paid sick days would be a good thing. But like, it's sort of, like farcical in a way. You know, for a lot of these women because there's no such thing as sick.

Indeed, many workers described being told explicitly they would lose their job if they called in sick. Others explained being penalized by temporary employment agencies by having income deducted from their pay.

### COVID-19-Related Health Problems and Impacts

Workers described the physical and mental impacts on their health of the pandemic. At the time of our study seven out of the 45 workers we interviewed had contracted COVID-19; however, the impact of the illness itself was minimally discussed by participants, none of whom had experienced severe health problems as a result. The fear of infection and the impact of the pandemic on employment and earnings were far more salient in workers’ accounts. For example, Afrin, 57-year-old, described how fear of infection in her day-to-day life impacted her:

When I am going to work, I feel extremely scared. As I am leaving the house, I get really scared. When I am on the subway, I feel scared. When I reach there [at the workplace], and I have to speak to someone or deal with a customer, that too feels scary. The whole time, it feels like I am in a climate of terror. I am fearful of what might happen, when or if I will get infected, I recite prayers on the way there and on the way back.

Interviews also highlighted the impacts of the pandemic on families, revealing an increase in household tensions as family members stayed home for lengthy periods of time, often in crowded environments. Amena, a community organizer, described the impact on women specifically:

Most of the pressure for the women because they have become sandwich[ed] right now. Husband wants to spend all their stress. Who is the person he can do it [to]? The wife. Children are angry. They want to show their anger. Who can they show it to? Their mother. I feel it like these women are in a situation they can be outburst any time. Sometimes outburst happens.

Importantly, COVID-19 added stress, fear, and another layer of uncertainty to the lives of workers who had already been carrying a heavy mental and physical health burden as a result of their precarious work experiences.

## Discussion

Our study brings to light the health and safety experiences of Bangladeshi immigrant workers during the second and third waves of the COVID-19 pandemic in Ontario. By and large, our findings are not unique to the Bangladeshi community, and instead reflect the experiences of other racialized immigrant workers in precarious employment. While the negative health and safety impacts of precarious work are well established, these impacts were heightened by the policies implemented by the federal and provincial governments in response to the public health emergency.

### Job Insecurity and Income Support

Our study details, among other findings, the ways in which workers shouldered economic impacts of the pandemic through increased employment and financial precarity and added costs. Other studies have documented increased job insecurity among immigrant workers in Canada compared to their Canadian-born counterparts in the early months of the pandemic,^63^ as well as a higher likelihood of reporting a financial impact from the pandemic and having applied for income support benefits as a result.^17^ Our study provides qualitative narratives of how this precarity was experienced by workers in their day-to-day lives, specifically the forms it took and the impacts it produced. Interview data indicate that the Canadian Emergency Response Benefit (CERB) was instrumental in helping many workers who moved out of work due to the pandemic; however, criteria such as the previous year income requirement negatively impacted eligibility for certain workers due to factors out of their control (e.g., recent immigration). The CERB ended in September 2020, as the second wave was gaining momentum, compelling many workers in our study to return to work despite the continued risk of exposure. While CERB was replaced with several new benefits, such as the Canada Recovery Benefit (CRB), the Canada Recovery Caregiving Benefit (CRCB), and the Canada Recovery Sickness Benefit (CRSB), these benefits were stricter in their eligibility requirements, required recipients to re-apply more frequently, entailed a lag between applying and receiving benefits, and, in the case of the CRB, had a lower benefit payout.^59^ These barriers effectively forced workers back into the workforce despite continued risks from COVID-19.

As previously discussed, the creation of the CERB was required largely due to an employment insurance (EI) system that failed to cover many workers as a result of decades of reforms that tightened eligibility requirements coupled with policies aimed at labour market flexibilization. Given the renewed focus on EI as a result of the pandemic, planned future EI reforms should aim to increase access by loosening eligibility requirements, account for the cost of living in determining income replacement levels, and further expand eligibility to include the myriad of reasons workers temporarily leave the workforce.^64^ Indeed, aside from unemployment, EI already works to cover parental leave, caregiver leave, and leave due to illness. Expanding and loosening eligibility requirements to expand coverage would make Canada better prepared for future public health crises.^65^

### Essential Work and Disposable Workers

In line with others that found that racialized and immigrant workers were over-represented in businesses or services that were designated as “essential” during the pandemic,^8,9^ many workers in our study were employed in essential work, all in front-line jobs.^f^ According to Statistics Canada's Labour Force Survey, the percentage of workers who worked from home was the lowest (17.2 percent) among those who made the lowest wages on an hourly basis. Working from home steadily increased with each increment in the hourly wage decile. Indeed, of the top 10 percent of wage-earners, 70.3 percent could work from home during the pandemic (based on Statistics Canada data for April 2020–June 2021 period).^63^ The same survey revealed the disproportionate burden of on-site work by workers from manufacturing, retail, and food and accommodation services.^66^ Worker participants in our study reflected this trend, as most were working minimum wage in one of these industries, and with none having the option to work from home during widespread lockdowns.

As noted by participants, the Ontario government's definition of essential work was broad. While the concept of essential work is not new in the Canadian context, the scope of work it covered was undeniably expanded during the COVID-19 pandemic. Our interviews highlight the fact that, taken together, the discourse and policies of essential work functioned as a coercive measure, rather than one of empowerment, running contrary to the role that it played in the public imagination (“clap for front-line workers,” “pandemic heroes,” etc.). By placing the emphasis on the essential nature of the work, rather than the worker, policies and measures surrounding essential work meant that the work must continue regardless of the conditions, essentially rendering those doing the work expendable. This was noted by some participants who reflected on the various manifestations of this lack of prioritization of their health and safety. As one worker noted, most essential workers in industries such as manufacturing, food, and retail, were eligible for vaccines in Phase 2 of Ontario's mass vaccination policy, unlike essential workers from the health care sector who were eligible in Phase 1.^67^ Given that these workers were similarly both compelled to continue working through their designation as essential and exposed to heightened health risks on the job due to COVID-19, the phased vaccine distribution created an unexplained hierarchy of essential workers. To address this, provincial guidelines should be established that clearly govern vaccine distribution by vulnerability and risk of exposure.

This lack of prioritization was further demonstrated by the fact that, while many participants’ work was deemed essential, they did not receive the pandemic pay meant to boost the wages of essential workers. As outlined in the Policy Context section above, the Ontario government chose to limit the distribution of federal funds to boost the pay of workers in the health and care sector. This policy choice excluded large numbers of workers, such as those in manufacturing, who were forced to continue to work despite increased risks. In light of this, provincial guidelines should be established that govern the designation of essential work during times of crises, including mandated temporary increases in pay for all workers deemed essential who face increased health and safety risks. In addition, the province should ensure, through proper inspections and monitoring systems, that businesses operating under an “essential work” designation are indeed essential.

Unsurprisingly, essential work has been associated with a significant increase in COVID-19 cases and deaths in Toronto.^68,69^ Workers reported that physical distancing was often impossible at work as conditions required proximity to others, often for extended periods. In the manufacturing sector, environmental controls, namely partitioning, was inconsistently used—though this measure may not even be appropriate in this setting, particularly in the absence of ventilation.^62^ Organizational factors increased exposure as workers reported crowding during shifts, at entry or exit points, and in break areas. While staggering shift and break times may help reduce crowding, these measures may conversely result in shorter shifts, and thus income loss, for workers. Our findings regarding conditions in the manufacturing sector reflect those of other studies, for instance in meatpacking, that found that the large number of workers per shift, the proximity of workers on processing lines, the prolonged duration of contact among workers, the enclosed nature of facilities, and the inconsistent or insufficient supplies of PPE put workers at high risk of contracting COVID-19.^70^

### Personal Protective Equipment

Similarly, our study documented a lack of available PPE. Existing research on COVID-19 and PPE has largely examined healthcare settings,^71–73^ while workers in other essential work categories have remained out of focus. Specifically, we found that workers’ health was placed at risk due to employers’ failure to provide PPE, such as N95 respirators. Consequently, workers procured their own masks, which were low cost, and re-used them. In Ontario, employers are legally required to provide workers PPE, however unlike some other Canadian jurisdictions, the legislation does not specify that it should provide it at no cost to the worker.^44^ Current City of Toronto and Ontario COVID-19 guidance for employers does not address the question of financial responsibility for PPE.^41,74^ Our results highlight the urgent need to provide high quality PPE at no cost to workers in low income, precarious work settings.

While PPE serves as a necessary measure, it should not be the sole focus, as it falls at the bottom of the hierarchy of controls and places the responsibility for health and safety on workers.^47^ Employers should adhere to the hierarchy of controls by implementing feasible engineering controls, such as improving ventilation systems, over solely resorting to PPE as a cost-effective, short-term measure to mitigate the spread of airborne hazards. The availability of PPE, in the presence of other strong infection control systems, have important implications for workers’ physical and mental health.^73,75^ Like other measures such as distancing and partitioning, PPE is most effective when combined with engineering controls such as good ventilation.^76^ However, workplaces may be reluctant to prioritize higher measures in the hierarchy due to the absence of mandates for the most effective measures such as ventilation. Consequently, adherence to public health guidelines may only marginally improve outcomes compared to noncompliance. This failure by the employer to put in place prevention measures in the workplace may be considered a violation of the general duty clause in occupational health legislation which mandates employers to “take all precautions reasonable in the circumstances to protect workers from being hurt or getting a work-related illness.”^46^ Therefore, it is imperative to institute and enforce strong public health regulations that incorporate the most effective control measures to ensure the safety of workers.

### Infection Management Gaps

Our interviews further documented infection management gaps, as workers reported a lack of openness from employers about potential exposures and questionable isolation practices and periods as a quick return to work was prioritized by employers concerned with production, and by workers who lacked the means to stay home. Workplace prevention and management gaps were reported to be particularly significant in the manufacturing sector, as client-facing sectors tended to follow public health regulations and guidelines more closely. It is worth noting that in Ontario, workers in manufacturing (31 percent) accounted for more workplace COVID-19 deaths (reported between April 2020 and December 2021) than any other sector, including health care (21 percent), as measured by workers’ compensation data.^77^ Unfortunately, due to lack of data regarding the number of employees at risk by industry, which would allow the calculation of rates, this information does not allow us to conclude that manufacturing was safer or less safe than other industries, despite what has been reported about different practices in risk prevention and management.

The lack of appropriate workplace monitoring was also raised by participants. During the pandemic, the Ontario government increased the number of occupational health and safety inspectors,^42^ and concentrated inspections in high-risk sectors such as manufacturing and warehousing.^43^ While inspections sometimes resulted in fines and shut downs,^78^ they generally focused on providing education and guidance to employers.^42^ Our results support the need for strengthened enforcement by allocating increased resources to the inspectorate and imposing greater penalties on employers.

### Paid Sick Leave

Our results also reinforce calls for a permanent paid sick leave program. As the workers in our study illustrate, the workers who lack paid sick leave are also those who need it the most. Namely, workers who lack paid sick leave are disproportionately working in low-income, precarious jobs on the front lines,^22^ such as the child care, food industry and factory workers in our study. These jobs tend to be disproportionately held by women, immigrant, migrants, racialized and Indigenous workers, raising important equity implications. In 2022, the federal government instituted ten paid sick days for federally regulated employees. Conversely, in Ontario, the newly elected Progressive Conservatives eliminated in 2018 two paid sick days that had been mandated by the previous government.^79^ During the pandemic, the provincial government put in place a temporary government-paid sick leave program that it subsequently extended until it was terminated on March 31, 2023. Workers and advocates have been calling for a permanent, employer-paid sick leave program that is permanent, universal (e.g., irrespective of employment or immigration status), hassle-free (no requirement for medical note and received in regular paycheques), adequate in terms of length, and fully paid.^80^ However, our results also indicate that unless the drivers of precarity are tackled, such as through increased regulation of temporary employment agencies, workers may not be able to take time off without jeopardizing their employment (as many workers are threatened with job loss for calling in sick which is against workers’ rights in Ontario) despite access to paid sick leave. Recommendations to reform temporary employment agencies and facilitate newcomers’ integration into decent work and are included in a report by the authors of this article. The report includes 55 recommendations aimed at reducing precarity and its negative consequences for workers. With regard to paid sick leave, the report recommends that the Government of Ontario establish a permanent employer-paid sick days program, providing 10 paid sick days to all workers at full wage.^81^

Taken together, our results demonstrate the significant work and health toll of the pandemic on precariously employed Bangladeshi immigrant workers in Toronto. In addition to work, workers experienced pandemic-related exposures and stressors at home and during transit. Indeed, in the early days of the pandemic, crowded transit was reported on routes that served large populations of essential workers in Toronto.^82^ As a result, workers described the heavy load of the pandemic on their physical and mental health. The impact was very much gendered, as women's traditional care-giving responsibilities were intensified during the pandemic, and as they absorbed stress and tension in the home.

Our results connect this disproportionate burden on health to systemic issues within the world of work and in policies and practices that create and perpetuate poverty and precarity. However, as noted previously, blame for the spread of infection has often been attributed to racialized immigrant communities’ “cultural practices,” such as gathering for religious celebrations and living in multigenerational homes,^33^ with solutions similarly focused on individual responsibility and behaviour. This public and political discourse and associated policies of personal responsibility during the pandemic was common within other largely racialized industries and workplaces, such as the agri-food industry and its large population of temporary foreign workers.^83^ Our study found no evidence that Bangladeshi workers’ lived experiences were shaped by their cultural practices or other community-specific factors, and instead provides support for the role of systemic factors in driving work and health inequities.

## Conclusion and Limitations

Although health and safety measures have been relaxed, the Toronto Bangladeshi community, as other racialized immigrant communities, continues to feel the pandemic's lasting impacts.^29^ Moreover, it is expected that pandemics of this magnitude will be increasingly frequent, highlighting the need for guidance around public health emergency preparedness and response. Our study proposed recommendations for governmental policy and practice and workplace organization and management on how to prepare and respond to infectious diseases public emergencies in work settings where workers experience multiple intersecting forms of vulnerability. This article represents an effort to amplify the voices of workers adversely impacted by the pandemic. Future studies should incorporate the perspective of employers to ensure that challenges to implementation of guidance and regulations are identified and addressed. As well, given that no worker in our study had submitted a workers’ compensation claim for COVID-19, our study could not examine experiences with workers’ compensation and return-to-work in this specific context. Future research should investigate access to workers’ compensation benefits and services and to health and safety rights for infectious diseases, including with regards to chronic impacts such as long COVID.

## Notes

a. This is the last date of available data.

b. The CERB was replaced by three new benefits, the Canada Recovery Benefit, the Canada Recovery Sickness Benefit, and the Canada Recovery Caregiving Benefit, each with its own eligibility requirement.

c. Formerly the South Asian Women’s Rights Organization (SAWRO).

d. Reflections on the community-engaged process are ongoing and will be written collectively for future publication.

e. In 2020, the minimum isolation period for COVID-19 symptomatic people were 10 days, until the new guideline reduced it to 5 days in December 2021.

f. Front-line jobs are defined here as jobs within essential industries that require employees to physically show up at work.
